# Photobiomodulation efficacy in age-related macular degeneration: a systematic review and meta-analysis of randomized clinical trials

**DOI:** 10.1186/s40942-024-00569-x

**Published:** 2024-08-15

**Authors:** Tiago N. O. Rassi, Lucas M. Barbosa, Sacha Pereira, Eduardo A. Novais, Fernando Penha, Luiz Roisman, Mauricio Maia

**Affiliations:** 1 Department of Ophthalmology, Banco de Olhos Foundation of Goiás, Goiânia, Brazil; 2 Department of Medicine, Federal University of Minas Gerais, Belo Horizonte, Brazil; 3 Department of Medicine, Faculty of Medical Science of Paraíba, João Pessoa, Brazil; 4 Department of Ophthalmology, Federal University of São Paulo, São Paulo, Brazil; 5 Department of Ophthalmology, Regional University of Blumenau, Blumenau, Brazil

**Keywords:** Age-related macular degeneration, Best-corrected visual acuity, Drusen volume, Geographic atrophy, Meta-analysis, Photobiomodulation

## Abstract

**Background:**

Age-related macular degeneration (AMD) is a leading cause of vision loss. Photobiomodulation (PBM) offers a controversial approach for managing dry AMD, aiming to halt or reverse progression through mitochondrial activity modulation. However, the efficacy and clinical relevance of PBM as a potential approach for managing dry AMD remain debated.

**Methods:**

We systematically searched PubMed, Embase, and Cochrane databases for randomized controlled trials (RCTs) comparing PBM versus a sham in patients with dry AMD. We performed trial sequential analysis (TSA) and minimal clinically important difference (MCID) calculations to assess statistical and clinical significance applying a random-effects model with 95% confidence intervals (CI).

**Results:**

We included three RCTs comprising 247 eyes. The pooled analysis showed that PBM significant improved BCVA (MD 1.76 letters; 95% CI: 0.04 to 3.48) and drusen volume (MD -0.12 mm³; 95% CI: -0.22 to -0.02) as compared with a sham control. However, the TSA indicated that the current sample sizes were insufficient for reliable conclusions. No significant differences were observed in GA area. The MCID analysis suggested that the statistically significant results did not translate into clinically significant benefits. In the quality assessment, all studies were deemed to have a high risk of bias.

**Conclusion:**

This meta-analysis points limitations in the current evidence base for PBM in dry AMD treatment, with issues around small sample sizes. Statistically significant improvements do not translate into clinical benefits. The research underscores need for larger RCTs to validate PBM’s therapeutic potential for dry AMD.

**Supplementary Information:**

The online version contains supplementary material available at 10.1186/s40942-024-00569-x.

## Background

Age-related macular degeneration (AMD) significantly impacts global visual health, particularly its advanced forms, such as geographic atrophy (GA), which leads to severe visual impairment and blindness. With population aging, the prevalence of AMD is expected to increase, highlighting the urgency for effective treatments and management strategies to mitigate its impact on quality of life and burden on healthcare systems [1]. 

Current therapeutic options for dry AMD are scarce and focus on lowering the progression to advanced stages such as GA, although their efficacy is often questionable [2]. Until recently, there were no treatments available specifically for GA. In 2023, the FDA approved two complement inhibitors for slowing the progression rate of GA areas [3, 4]. However, accessibility remains a major challenge. This underscores the critical need for novel therapies that can halt or ideally reverse the progression of dry AMD and GA, thereby preserving visual function.

Photobiomodulation (PBM) is a therapeutic option for dry AMD, focusing on slowing disease progression by influencing mitochondrial activity, reducing oxidative stress, and modulating inflammation through LEDs at specific wavelengths (590, 660, 850 nm) [5, 6]. Despite anecdotal reports and early studies indicating potential benefits, such as improved microperimetry outcomes for some patients, [7] its efficacy and scientific validity in preventing the progression from dry AMD to GA are subject of substantial controversy [8, 9].

Herein, we perform an updated meta-analysis of randomized controlled trials (RCTs) to evaluate the efficacy of PBM versus a sham procedure in patients with dry AMD. We performed a trial sequential analysis (TSA) to evaluate if the sample was sufficient for making statistical inference [10–12] and assessed the minimum clinically important differences (MCID) calculated by pooled standard deviation (SD) to check if any statistical differences would translate to clinical significance [13, 14]. 

## Methods

Our study was performed and reported following the Cochrane Collaboration Handbook for Systematic Reviews of Interventions and the Preferred Reporting Items for Systematic Reviews and Meta-Analysis (PRISMA) Statement guidelines [15, 16]. The protocol was prospectively registered in the International Prospective Register of Systematic Reviews (PROSPERO) under protocol number CRD42024521983.

### Data source and search strategy

We systematically searched PubMed, Embase, and Cochrane databases. Our search was last updated in February 2024. The search terms included “photobiomodulation” and “age-related macular degeneration”. The complete search strategy is provided in Supplemental Methods 3. All records retrieved were independently assessed by two authors, L.M.B. and T.N.O.R., and a decision regarding full-text retrieval was arbitrated by consensus between them. Full texts were reviewed by L.M.B. and T.N.O.R. and discussed regarding inclusion and exclusion criteria. References of eligible papers and systematic reviews were also searched for additional studies of interest. Conference abstracts and prospective trials were also searched.

### Eligibility criteria

There was no restriction regarding publication date, status, or language. We considered studies eligible for inclusion if they [1] were RCTs; [2] directly compared PBM with sham; [3] included patients with diagnosed non-exudative AMD.

### Endpoints

Our clinical outcomes of interest were: [1] last visit best corrected visual acuity (BCVA); last visit drusen volume in mm^3^; last visit GA area in mm^2^. Our pooled analyses last visit included a follow-up of at least 9 months.

### Risk of bias assessment

Two independent authors (TR. and S.F.P.) assessed the risk of bias in the included RCTs using the Cochrane tool for assessing the risk of bias in randomized controlled trials (RoB-2) [17]. Disagreements were resolved through consensus.

### Statistical analysis

We applied the Mantel-Haenszel random-effects model with a restricted maximum likelihood variance estimator for all outcomes. We pooled risk ratios (RR) with 95% confidence intervals (CI) for binary endpoints and mean differences (MD) with 95% CI for continuous endpoints. When needed, we extracted data using the WebPlotDigitizer tool.

We assessed heterogeneity with Cochran’s Q and I^2^ statistics, with *p* ≤ 0.10 indicating statistical significance for heterogeneity. We determined the between-study heterogeneity based on I^2^ values of 0%, ≤ 25%, ≤ 50%, and > 50%, indicating no observed, low, moderate, and substantial heterogeneity, respectively. All statistical analyses were performed using R version 4.3.2.

### Trial Sequential Analysis

We performed TSA using the TSA software (Copenhagen Trial Unit, Centre for Clinical Intervention Research, Copenhagen, Denmark) on the outcomes of BCVA, drusen volume, and GA area. We utilized a MD measure of effect and a random-effects model, setting a conventional 95% CI. The analysis incorporated a two-sided conventional boundary with 5% types I error rate. Alpha-spending boundaries were established using a two-sided boundary type, maintaining a 5% types I error rate and an 80% statistical power. The alpha and beta spending function adopted was the O’Brien-Fleming approach. In determining the required information size (RIS), we opted for an empirical method with heterogeneity correction, applying the model variance to accommodate study variability.

### Minimal clinically important difference

We established the MCID for each outcome exhibiting statistical differences by calculating the pooled standard deviation (SD) and then multiplying this pooled SD by 0.5 [13, 14, 18]. 

## Results

### Study selection and characteristics

**Fig. 1:**
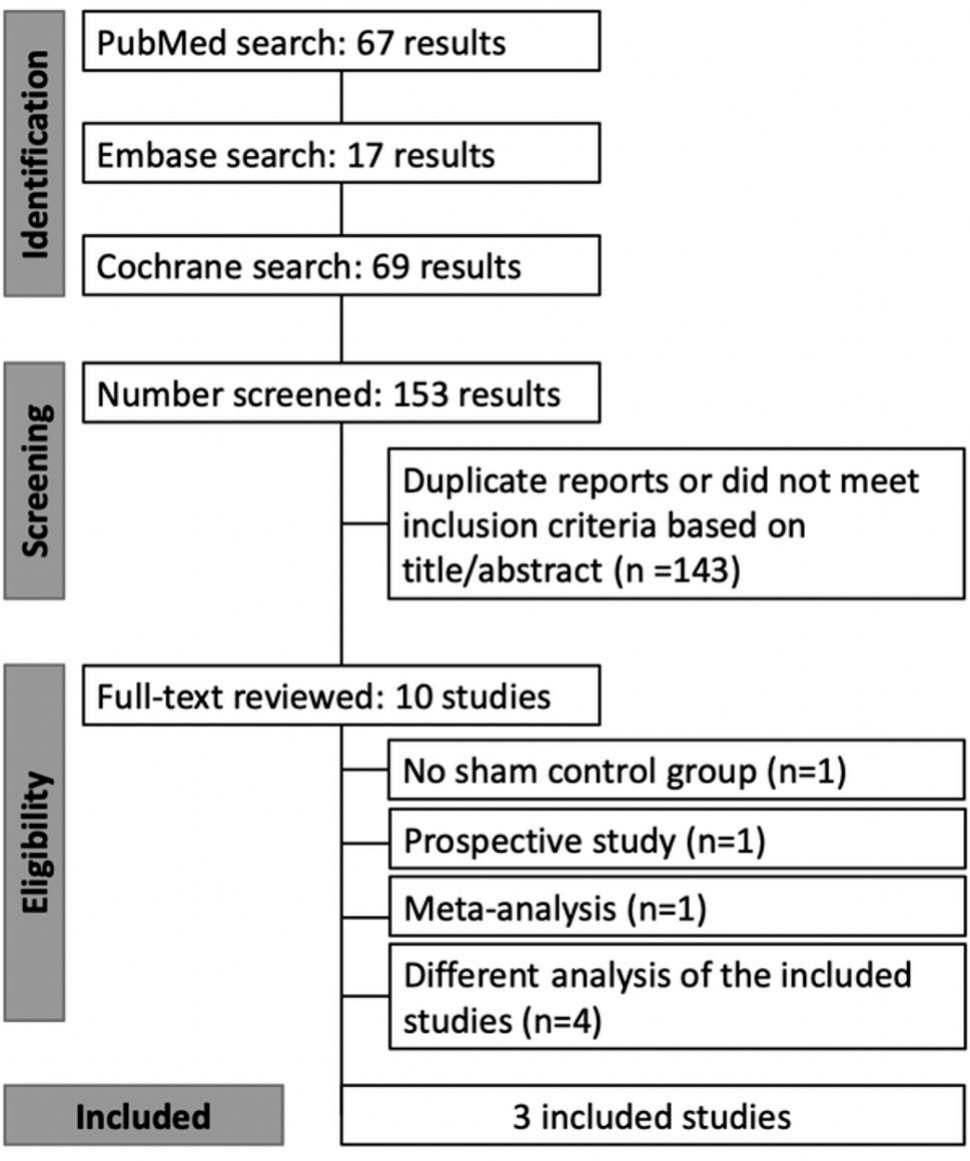
PRISMA flow diagram of study screening and selection. *Abbreviations* PBM, photobiomodulation; PRISMA, Preferred Reporting Items for Systematic Reviews and Meta-Analysis

Our systematic review initially yielded 150 results. After removal of duplicates and screening based on title and abstract, 10 full-text articles were reviewed for possible inclusion. Finally, three RCTs fulfilled our inclusion criteria and were included in the analysis, [7, 19, 20] comprising a pooled population of 247 eyes, of whom 151 (61%) were randomized to the PBM group. Comprehensive details of the study selection are detailed in Fig. 1.

The mean age was 75.1 years. Total follow-up ranged from 9 to 13 months. All included studies were sham-controlled. Individual study characteristics are detailed in Table 1 [7, 19, 20]. 

**Table 1:** Baseline characteristics of included studies

Study	Study Design	Location	No. of Patients	Eyes	Eyes PBM/Sham	Age (SD)	Follow-up (months)	Primary Endpoint	AREDS 1/2/3/4
Boyer [19]	Double-masked, randomized, sham-controlled, parallel group, multicenter prospective study	USA	100	148	93/55	75.4 (7.1)	13	BCVA	0/19/126/0
Burton [20]	Prospective, randomized, double-masked clinical trial	Europe	44	53	34/19	74.1 (8.0)	9	BCVA	1/11/35/6
Markowitz [7]	Double-masked, randomized, sham-controlled, parallel group	Canada	30	46	24/22	76 (8.3)	12	BCVA	0/1/14/31

* Abbreviations* AREDS, Age-Related Eye Disease Studies; BCVA, best-corrected visual acuity; No., number; SD standard deviation, USA, United States of America

### Clinical endpoints

**Fig. 2:**
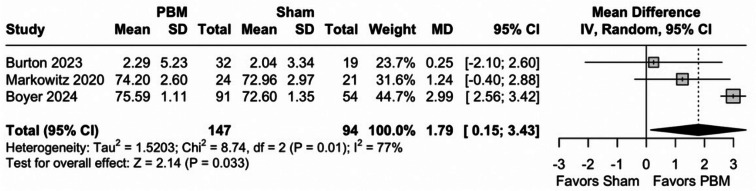
Forest plot for best corrected visual acuity (BCVA). There was a slight overall improvement favoring PBM versus sham with a mean difference of 1.76 (*P* = 0.04). *Abbreviations* CI, confidence intervals; MD, mean difference; PBM, photobiomodulation; SD, standard deviation; IV, inverse variance

**Fig. 3:**
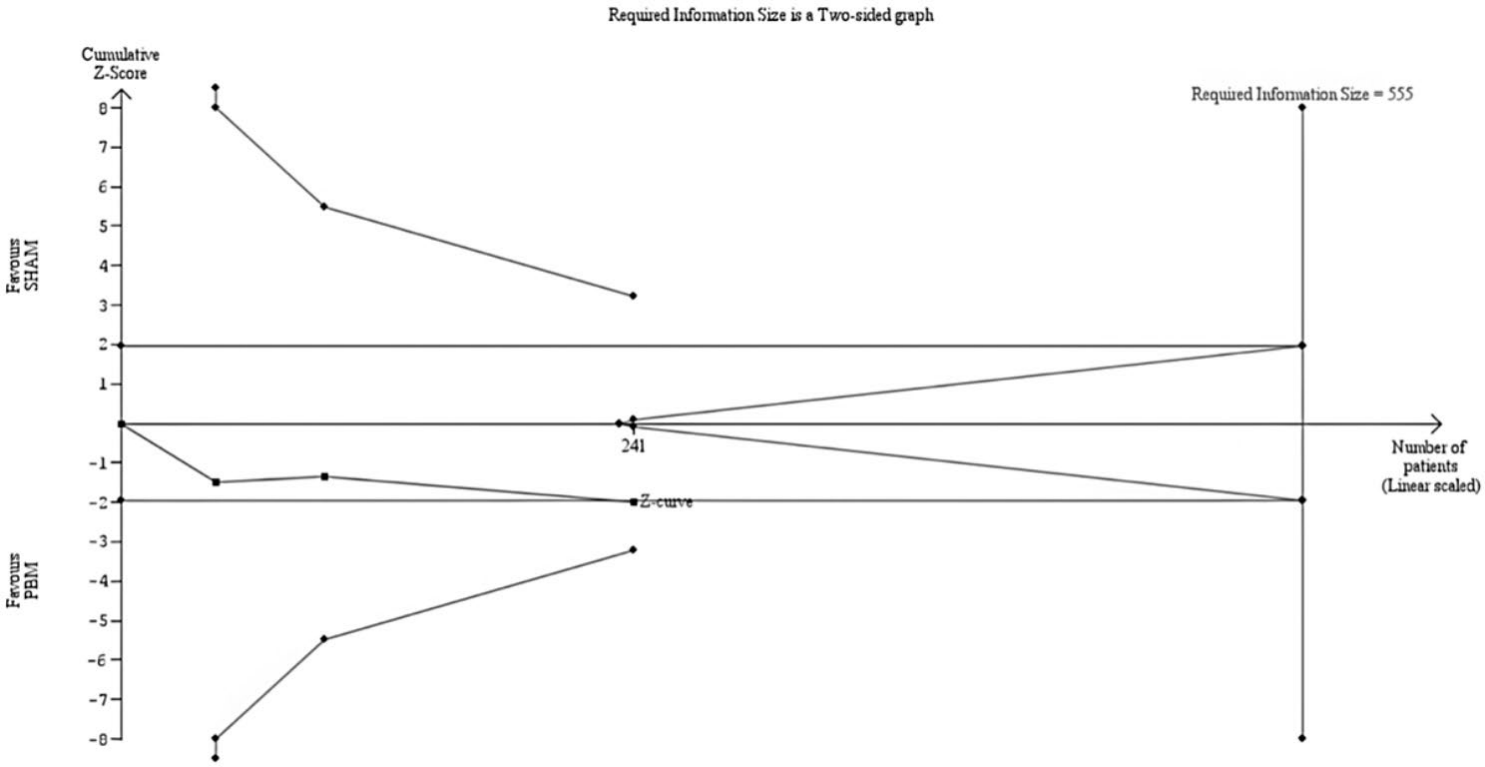
Figure 3 shows a TSA for evaluating treatment efficacy in a cumulative meta-analysis. On the x-axis, the number of eyes reaches 241 across 3 studies, as shown by the blue curve. The y-axis measures the Z-score, assessing statistical deviation from the null hypothesis. The curve falls short of the required information size (555 eyes), indicated by the perpendicular line, suggesting more data is needed for a robust conclusion. The curve does not cross the monitoring boundaries, which, along with the conventional ± 1.96 Z-score boundaries, assess significance; therefore, the analysis does not conclusively favor either treatment group over the other

PBM showed a statistically significant improvement in BCVA over sham treatment, with a MD of 1.76 ETDRS letters among 241 eyes (95% CI [0.04; 3.48], *p* = 0.04) despite a high heterogeneity (I²=77%), as shown in Fig. 2. However, while statistically significant, the observed improvement did not meet the threshold for clinical relevance as defined by the MCID of 6.8 ETDRS letters. The TSA also indicated a RIS of 555 eyes for statistical inference, as the z-curve did not meet the monitoring boundary, suggesting the current sample size is insufficient, as shown in Fig. 3.

### Anatomical endpoints

**Fig. 4:**
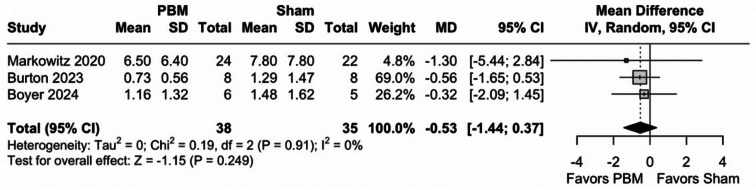
Forest plot for of geographic atrophy (GA) area between Photobiomodulation (PBM) and sham treatment. The combined results yield a mean difference of -0.53, indicating no significant difference between PBM and SHAM treatments in reducing GA area (*P* = 0.25). *Abbreviations* CI, confidence intervals; MD, mean difference; PBM, photobiomodulation; SD, standard deviation; IV, inverse variance

**Fig. 5:**
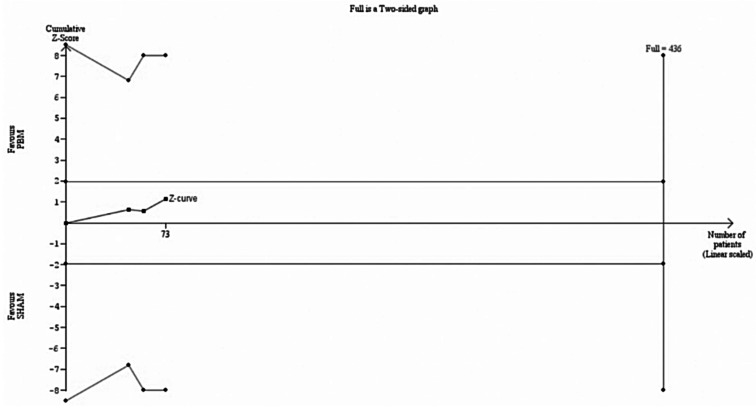
TSA for GA area. On the x-axis, the number of eyes reaches 73 across 3 studies, as shown by the blue curve. The y-axis measures the Z-score, assessing statistical deviation from the null hypothesis. The curve falls short of the required information size (436 eyes), suggesting that more data are needed for a robust conclusion. The curve does not cross the monitoring boundaries, which, along with the conventional ± 1.96 Z-score boundaries, assess significance; therefore, the analysis does not conclusively favor either treatment group over the other. *Abbreviations* PBM, photobiomodulation

There was no significant difference between groups in GA area (73 eyes; MD -0.53 mm^2^; 95% CI [-1.44; 0.37]; *p* = 0.25; I^2^ = 0%), as shown in Fig. 4. However, TSA indicated that a RIS of 436 eyes would be necessary for a statistical inference, as shown in Fig. 5. Moreover, the z-curve did not reach the monitoring boundary.

**Fig. 6:**
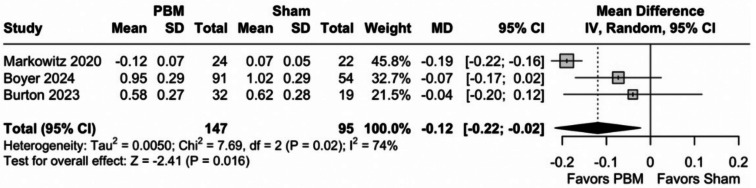
Forest plot for drusen volume. Results show a small mean difference of -0.12 mm³, with overall findings favoring PBM (*P* = 0.02). *Abbreviations* CI, confidence intervals; MD, mean difference; PBM, photobiomodulation; SD, standard deviation; IV, inverse variance

**Fig. 7:**
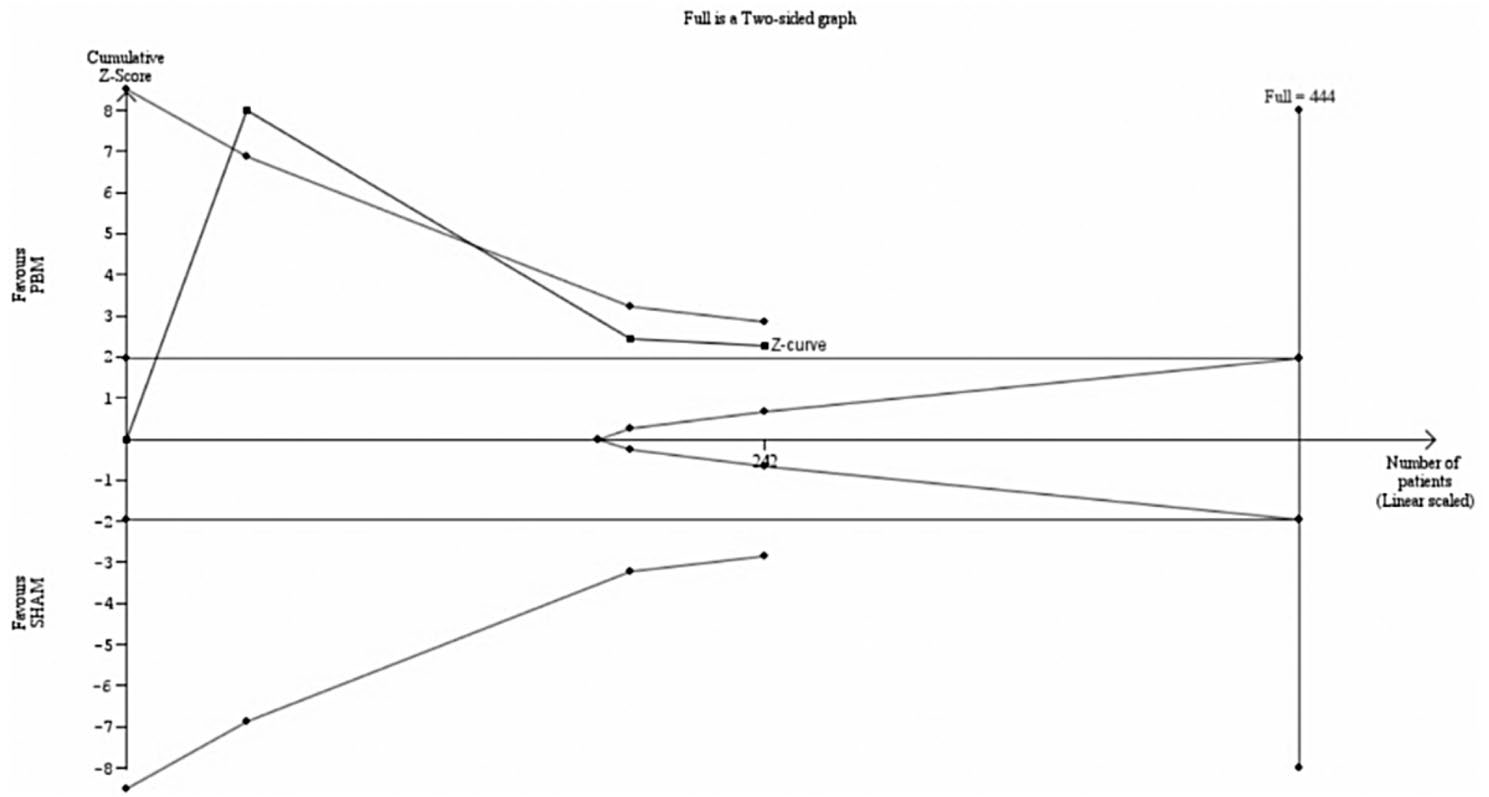
TSA for drusen volume. On the x-axis, the number of eyes reaches 242 across 3 studies, as shown by the blue curve’s progression. The y-axis measures the Z-score, assessing statistical deviation from the null hypothesis. The curve falls short of the required information size (444 eyes), indicated by the perpendicular line, suggesting more data are needed for statistical inference. The curve does not cross the monitoring boundaries, which, along with the conventional ± 1.96 Z-score boundaries, assess significance; therefore, the analysis does

As compared with a sham procedure, PBM significantly reduced drusen volume (242 eyes; MD -0.12mm^3^; 95% CI [-0.22; -0.02]; *p* = 0.02; I^2^ = 74%), as shown in Fig. 6. However, the observed improvement did not meet the threshold for clinical relevance as defined by the MCID of 0.39 mm^3^. In addition, TSA indicated that a RIS of 444 eyes would be necessary for statistical inference, indicating insufficient sample size, as shown in Fig. 7. Moreover, the z-curve did not reach the monitoring boundary.

### Risk of Bias Assessment

**Fig. 8:**
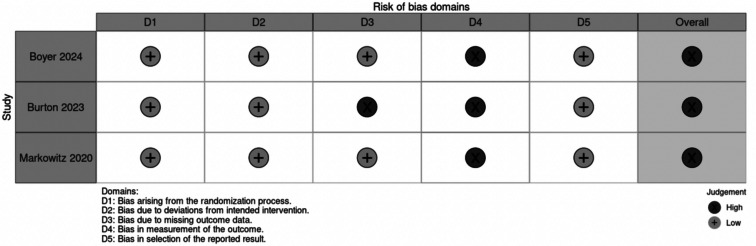
Risk of bias assessment

Using the Cochrane Collaboration’s RoB-2 tool, our quality assessment suggests that all three RCTs are at a high risk for bias. The primary concern was related to bias in measuring outcomes. Additionally, one of the studies experienced issues with bias due to missing outcome data attributed to disruptions caused by COVID-19 [20]. Individual RCT appraisal is detailed in Fig. 8.

## Discussion

This meta-analysis included three RCTs with 247 eyes to assess the efficacy of PBM in patients with dry AMD. Our pooled data showed an improvement in BCVA and drusen volume in patients treated with PBM as compared with a sham with no significant difference in terms of progression of GA area. However, these statistical inferences could not be confirmed due to insufficient sample size, as indicated by the TSA. Even if the TSA was favorable, BCVA and drusen volume were not clinically significant, as they did not meet the MCID.

One may argue that the significant findings of RCTs of PBM therapy for dry AMD may not translate into clinical benefits. The largest RCT on the subject found a MD of 2.4 ETDRS letters compared with sham [19]. Nonetheless, visual acuity measurements in intermediate AMD may vary by an average of 9 ETDRS letters in patients who do not receive any treatment, much higher than the above cited MD [21]. For instance, the established MCID for photodynamic therapy in patients with neovascular membranes is 7.5 letters [22]. Of note, the FDA requires a minimum improvement of at least 15 letters for approving a pharmacological intervention in this setting [23]. Therefore, it could be contended that the benefits of PBM therapy do not meet clinical significance, which indeed was corroborated by our findings through the MCIDs results.

In addition, inadequate sample sizes limit the primary studies from definitively assessing the efficacy of PBM for dry AMD, as highlighted by previous meta-analyses that were only able to collect data from 2 studies and 96 eyes [8]. The individual trials, LIGHTSITE I and II, [7, 20] also recognized the constraints of their small cohorts. On top of the limited sample size, there are only three RCTs evaluating PBM for dry AMD, highlighting the need for additional and larger RCTs. Additionally, some might argue that the pooled sample size lacked statistical power for measuring outcomes such as the drusen volume. This issue arises because drusen size may vary, and drusen regression is a well-described phenomenon in the natural course of the disease, [24, 25] underscoring the need for larger sample sizes to draw more robust conclusions [26]. All these data and insights were corroborated by our TSA, which revealed that the existing pooled sample did not meet the required information size to make statistical inferences.

A significant challenge in evaluating treatments for dry AMD is selecting appropriate clinical endpoints. The FDA only recognizes GA volume as a valid outcome for dry AMD, whereas visual acuity and changes in drusen volume are not accepted by the regulatory agency [3]. This obstacle in finding appropriate measuring outcomes may explain the barriers that current studies on PBM face when trying to assess treatment efficacy in dry AMD. This is reflected heavily in the quality assessment, where all the studies were deemed to be at high risk of bias, consistent with evaluation of a previous meta-analysis [8]. One of the reasons for this high risk of bias was the reliance on BCVA as a measure of efficacy.

It is highly questionable whether BCVA stands as an optimal measure for treatment efficacy for drusen, since visual acuity may not be sensitive enough to detect changes in visual function in patients with intermediate AMD [27]. Another study showed lack of correlation between large drusen and BCVA [28]. Visual acuity has also shown major variations in intermediate AMD, which could potentially interfere with results. [21].

Additionally, the application of short-term drusen volume tracking as an effective endpoint for assessing efficacy in AMD has its restrictions. Studies with extended durations have demonstrated that a reduction in drusen can actually be indicative of a risk for progressing to advanced stages of AMD [3, 24, 25].

Our study has limitations. First, the small size of our pooled population may have hindered our statistical power, despite the inclusion of all studies that met eligibility criteria. Second, the absence of patient-level data precluded assessment of subgroup analyses and whether individual factors may interfere in the relative efficacy of PBM in this patient population. Finally, we could not assess the incidence of new-onset GA owing to the incomplete reporting in some of the individual studies.

## Conclusion

In this meta-analysis evaluating PBM therapy for patients with dry AMD, there was a statistically significant improvement in visual acuity and drusen volumes, but not in incidence of GA. However, definitive statistical inferences are limited by an insufficient sample size, as indicated by the TSA. In addition, the significant results in terms of visual acuity and drusen volumes did not translate into clinically important benefits, as they did not meet the MCID casting doubt on PBM’s real-world efficacy. Larger RCTs with longer follow ups and more appropriate outcome measures are warranted to conclusively evaluate the role of PBM in patients with dry AMD.

### Electronic supplementary material

Below is the link to the electronic supplementary material.

Supplementary Material 1

## Data Availability

No datasets were generated or analysed during the current study.

## Abbreviations

AMD age: Related macular degeneration
AREDS age: Related eye disease studies
ARMD age: Related macular degeneration
BCVA best: Corrected visual acuity
CI: Confidence intervals
ETDRS: Early treatment of diabetic retinopathy study
FDA: Food and Drug Administration
GA: Geographic atrophy
MCID: Minimum clinically important differences
MD: Mean difference
PBM: Photobiomodulation
PRISMA: Preferred Reporting Items for Systematic Reviews and Meta-Analysis
PROSPERO: International Prospective Register of Systematic Reviews
RCT: Randomized controlled trial
RIS: Required information size
RoB-2: Risk of Bias Assessment Tool 2
SD: Standard deviation
TSA: Trial sequential analysis
